# Supplementary material for: Epigenetic regulation of CD38/CD48 by KDM6A mediates NK cell response in multiple myeloma
Source: Nat Commun. 2024 Feb 14;15:1367. doi: 10.1038/s41467-024-45561-z (PMC10866908; doi:10.1038/s41467-024-45561-z)
Supplement: Supplementary file 1 — Supplementary Information [file 41467_2024_45561_MOESM1_ESM.pdf]

Supplementary Information

Epigenetic regulation of CD38/CD48 by KDM6A mediates NK cell response in multiple myeloma

Jiye Liu[1], Lijie Xing[2], Jiang Li[3], Kenneth Wen[1], Ning Liu[1,4], Yuntong Liu[1], Gongwei Wu[5], Su Wang[6], Daisuke Ogiya[7], Tian-Yu Song[8,9], Keiji Kurata[1], Johany Penailillo[10], Eugenio Morelli[1], Tingjian Wang[11], Xiaoning Hong[3], Annamaria Gulla[12,1], Yu-Tzu Tai[1], Nikhil Munshi[1], Paul Richardson[1], Ruben Carrasco[10,13], Teru Hideshima[1], Kenneth C. Anderson[1,*]

Affiliations

[1]Jerome Lipper Multiple Myeloma Center, Lebow Institute for Myeloma Therapeutics, Department of Medical Oncology, Dana-Farber Cancer Institute, Boston, MA 02215, USA.

[2]Department of Hematology, Shandong Cancer Hospital and Institute, Shandong First Medical University and Shandong Academy of Medical Sciences, Jinan, Shandong 250117, China.

[3]Clinical Big Data Research Center, The Seventh Affiliated Hospital of Sun Yat-Sen University, Shenzhen, Guangdong 518107, China.

[4]Department of Marine Bio-Pharmacology, College of Food Science and Technology, Shanghai Ocean University, Shanghai 201306, China.

[5]Center for Functional Cancer Epigenetics, Department of Medical Oncology, Dana-Farber Cancer Institute, Boston, MA 02215, USA.

[6]Vertex pharmaceuticals, Boston, MA 02210, USA.

[7]Department of Hematology and Oncology, School of Medicine, Tokai University, Isehara 259-1193, Japan.

[8]Department of Medical Oncology, Dana-Farber Cancer Institute, Boston, MA 02215, USA.

[9]Broad Institute of Harvard and MIT, Cambridge, MA 02142, USA.

[10]Department of Oncologic Pathology, Dana-Farber Cancer Institute, Boston, MA 02215, USA.

[11]Department of Cancer Biology, Dana-Farber Cancer Institute, Boston, MA 02215, USA.

[12]Candiolo Cancer Institute, FPO-IRCCS-Candiolo (TO) 10060, Italy.

[13]Department of Pathology, Brigham and Women's Hospital, Harvard Medical School, Boston, MA 02215, USA

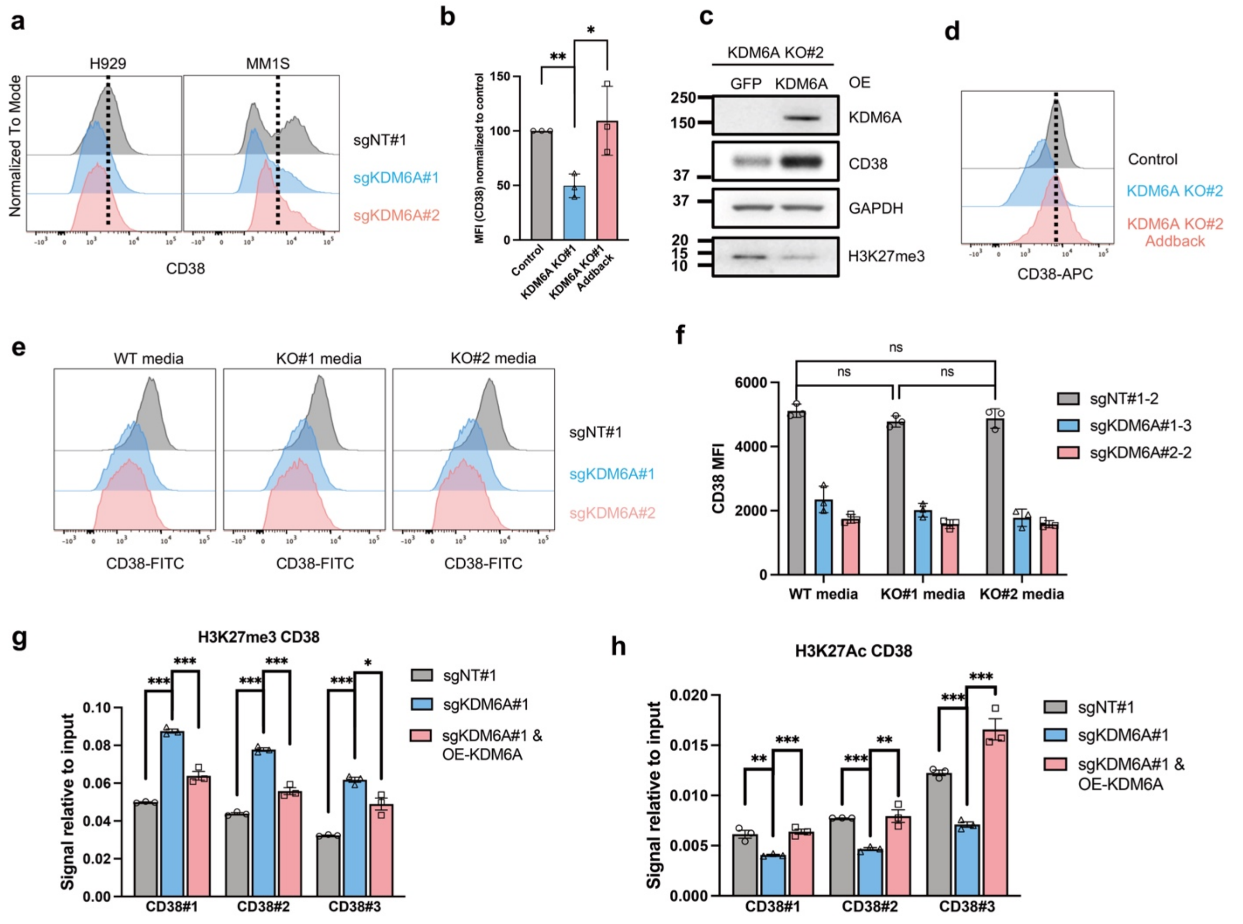

Supplementary Fig. 1. KDM6A regulates CD38 expression at the transcriptional level. a, Representative flow cytometry analysis of CD38 expression in H929 and MM1S transfected with indicated sgRNAs. **b**, Mean fluorescence intensity (MFI) of CD38 after ectopic overexpression of KDM6A in *KDM6A* KO H929 cells (mean ± SEM, n=3 biologically independent experiments). *p<0.05; **p<0.01 (two-sided student's t test). **c**, Western blotting of CD38 protein levels after ectopic overexpression of KDM6A in KDM6A KO H929 cells. OE, overexpression. Three independent experiments were performed and similar results were obtained. **d**, Representative flow cytometry analysis of CD38 expression level after ectopic overexpression of KDM6A in KDM6A KO H929 cells. **e**,**f**, Representative flow cytometry analysis (**e**) and relative MFI value (**f**) of CD38 expression

in KDM6A WT or KO cells cultured in KDM6A WT or KO cells medium. ns, not significant. **g**,**h**, H3K27me3 (**g**) and H3K27Ac (**h**) ChIP-qPCR analysis at the CD38 gene in KDM6A WT, KO, and KDM6A add-back H929 cells (mean ± SEM, n=3 biologically independent experiments). *p<0.05; ***p<0.001 (two-sided student's *t* test). Source data are provided as a Source Data file.

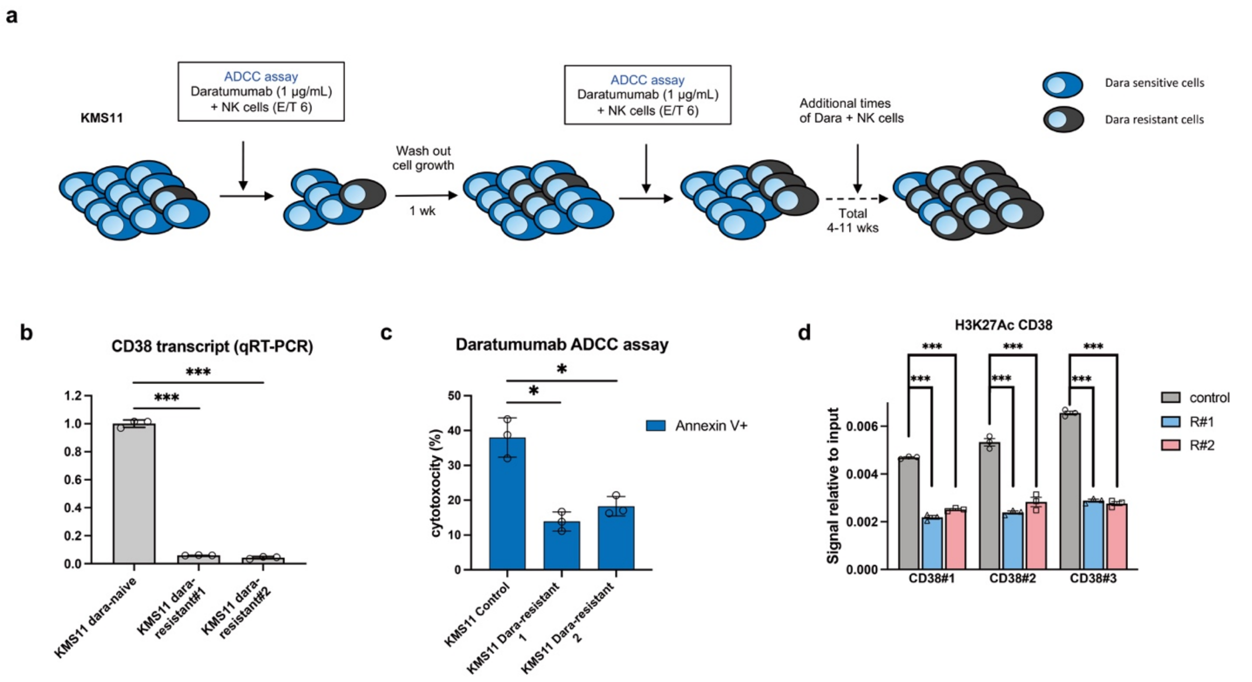

Supplementary Fig. 2. Establishment of Dara-resistant MM cell line. a, Schematic design of generation of Dara-resistant MM cell line. **b**, q-RT-PCR for CD38 mRNA in KMS11 Dara-sensitive- and resistant-cells. Data were normalized against GAPDH (mean ± SEM, n=3 biologically independent experiments). ***p<0.001 (two-sided student's t test). **c**, KMS11 Dara-sensitive- and resistant cells were co-cultured with primary human NK cells and Dara and subjected to ADCC assay. The cells were stained with Annexin V and 7AAD and analyzed by flow cytometry (mean ± SEM, n=3 biologically independent experiments). *p<0.05 (two-sided student's t test). **d**, H3K27Ac ChIP-qPCR analysis at the CD38 gene in KMS11 Dara-sensitive- and resistant cells (mean ± SEM, n=3 biologically independent experiments). ***p<0.001 (two-sided student's t test). Source data are provided as a Source Data file.

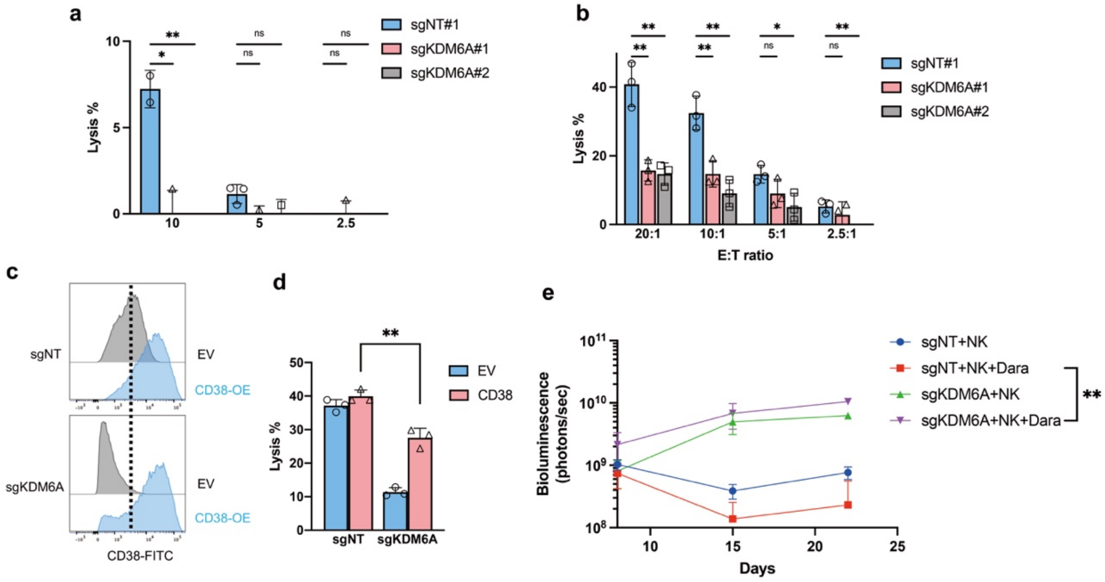

Supplementary Fig. 3. *KDM6A* KO renders MM cells resistant to Dara-mediated ADCC through CD38 downregulation. a, *KDM6A* WT and KO MM1S cells were co-cultured with primary human NK cells and Dara and subjected to ADCC assay (mean ± SEM, n=3 biologically independent experiments). ns, not significant; *$p<0.05$; **$p<0.01$ (two-sided student's *t* test). **b**, *KDM6A* WT and KO H929 cells were co-cultured with PBMC and Dara and subjected to ADCC assay (mean ± SEM, n=3 biologically independent experiments). ns, not significant; *$p<0.05$; **$p<0.01$ (two-sided student's *t* test). **c**, Representative flow cytometry analysis of CD38 expression level after ectopic overexpression of CD38 in KDM6A WT or KO H929 cells. EV, empty vector. **d**, ADCC assay of *KDM6A* KO and WT H929 cells after ectopic overexpression of CD38 and co-cultured with primary human NK cells and Dara (mean ± SEM, n=3 biologically independent experiments). **$p<0.01$ (two-sided student's *t* test). **e**, Quantification of tumor growth expressed as average bioluminescence signal of mice transplanted with *KDM6A* KO or WT H929 cells and treated with human primary NK cells or NK+Dara (8 mg/kg). **$p<0.01$ (two-sided student's *t* test). Source data are provided as a Source Data file.

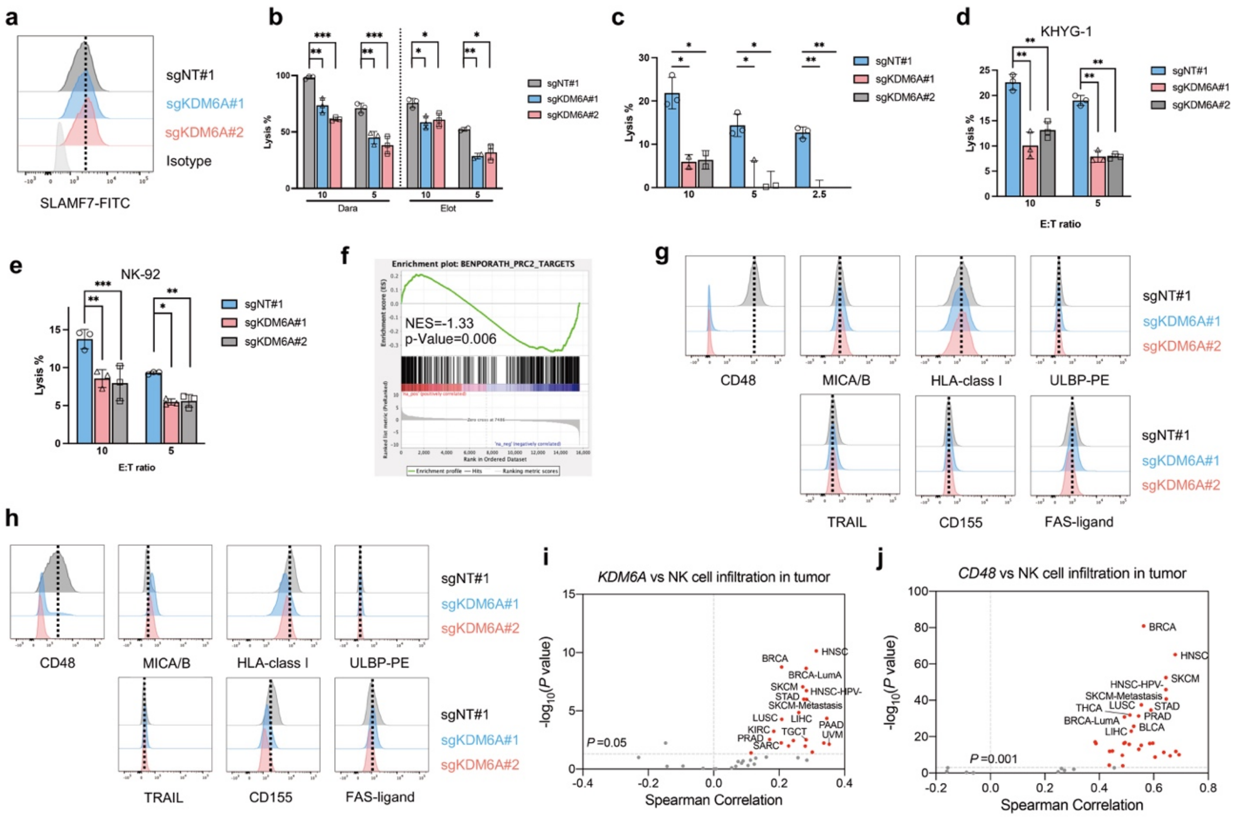

Supplementary Fig. 4. KDM6A regulates CD48 expression in MM cells. a, Representative flow cytometry analysis of SLAMF7 expression in H929 cells transfected with indicated sgRNAs. **b**, *KDM6A* WT and KO H929 cells were co-cultured with primary human NK cells and Dara or Elotuzumab (Elot), and subjected to ADCC assay (mean ± SEM, n=3 biologically independent experiments). *p<0.05; **p<0.01; ***p<0.001 (two-sided student's t test). **c**, Normalized lysis percentage of KDM6A WT or KO MM1S cells under the cocultivation with primary NK cells at different E:T ratios with IL-2 for 6 hours (mean ± SEM, n=3 biologically independent experiments). *p<0.05; **p<0.01 (two-sided student's t test). **d**,**e**, Normalized lysis percentage of KDM6A WT or KO H929 cells under the cocultivation with KHYG-1 (**d**) or NK-92 (**e**) cells at different E:T ratios with IL-2 for 6 hours (mean ± SEM, n=3 biologically independent experiments). *p<0.05; **p<0.01; ***p<0.001 (two-sided student's t test). **f**, Gene set enrichment analysis plots of datasets

identified comparing *KDM6A* KO and wild-type signatures. NES, normalized enrichment score. **g**,**h**, Representative flow cytometry analysis of indicated surface protein expression level in KDM6A WT and KO H929 (**g**) or MM1S (**h**) cells. **i**,**j**, KDM6A (**i**) and CD48 (**j**) mRNA levels correlate with primary NK cell infiltration in indicated cancers. Volcano plot showing the Spearman's correlation and estimated significance of KDM6A or CD48 with NK cell infiltration from RNA-seq data across TCGA cancer types calculated by TIMER (Tumor Immune Estimation Resource) and adjusted for tumor purity. Each dot represents a cancer type in TCGA; red dots indicate significant correlations ($p < 0.01$). Source data are provided as a Source Data file.

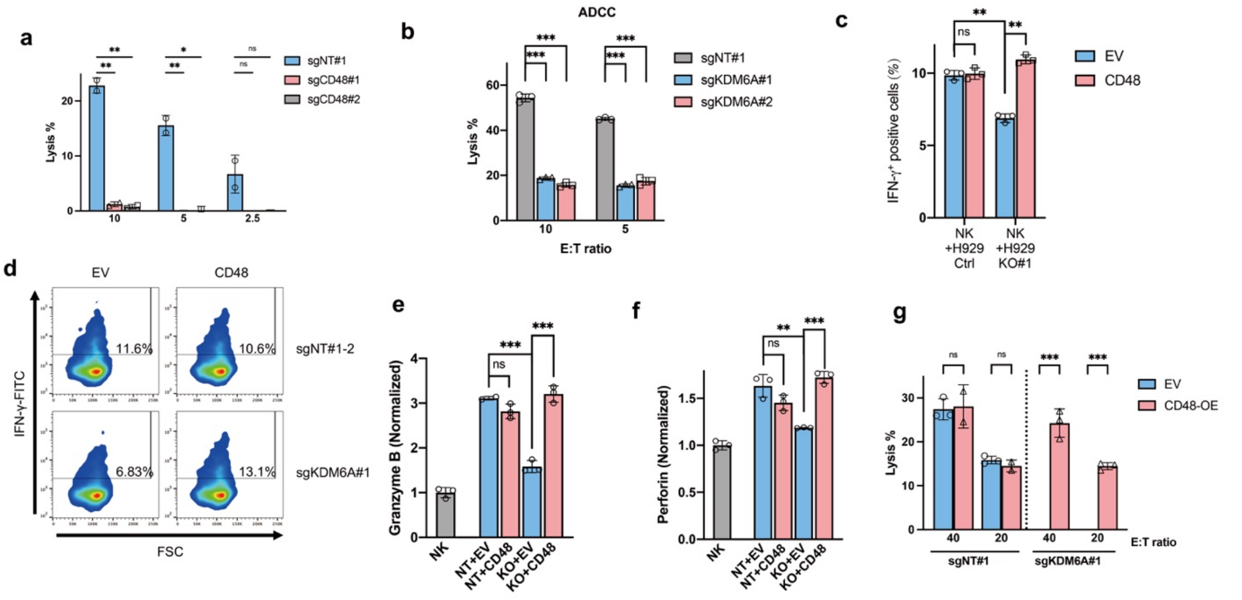

Supplementary Fig. 5. KDM6A mediates NK activity through CD48 regulation in MM cells. a, Normalized lysis percentage of CD48 WT or KO H929 cells under the cocultivation with primary NK cells at different E:T ratios with IL-2 for 6 hours (mean ± SEM, n=2 biologically independent experiments). ns, not significant; **p*<0.05; ***p*<0.01 (two-sided student's *t* test). **b**, KDM6A WT and KO H929 cells were co-cultured with primary human NK cells and Dara at different E:T ratios, and subjected to ADCC assay (mean ± SEM, n=3 biologically independent experiments). ****p*<0.001 (two-sided student's *t* test). **c**, Intracellular IFN-γ staining of primary NK cells co-cultured with KDM6A WT or KO H929 cells after ectopic overexpression of CD48 for 6 hours (mean ± SEM, n=3 biologically independent experiments). ns, not significant; ***p*<0.01 (two-sided student's *t* test). **d**, Representative flow cytometry analysis of IFN-γ in (**c**). **e**,**f**, *KDM6A* KO or control H929 cells after ectopic overexpression of CD48 were co-cultured with primary NK cells for 6 hours and the supernatant was collected for granzyme B (**e**) and perforin (**f**) ELISA assay (mean ± SEM, n=3 biologically independent experiments). ns, not significant; ***p*<0.01; ****p*<0.001 (two-sided student's *t* test). **g**, ADCC assay of *KDM6A*

KO and WT H929 cells after ectopic overexpression of CD48 and co-cultured with PBMC and Dara at different E:T ratios (mean ± SEM, n=3 biologically independent experiments). ns, not significant; ***$p<0.001$ (two-sided student's *t* test). Source data are provided as a Source Data file.

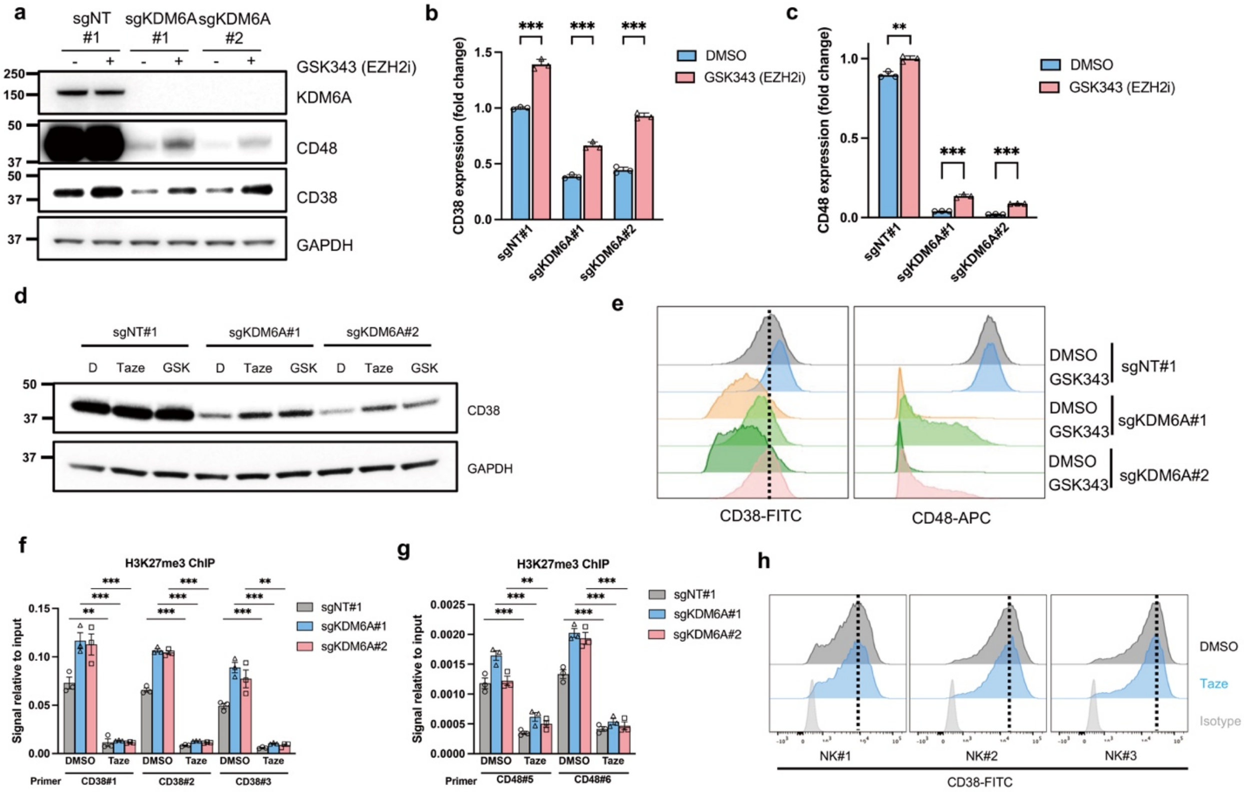

Supplementary Fig. 6. EZH2 inhibitors increase CD38/CD48 expression. a, Western blotting of CD38 and CD48 protein levels in KDM6A KO and control H929 cells treated with GSK343 (4 μM) for 4 days. Three independent experiments were performed and similar results were obtained. **b**,**c**, q-RT-PCR for CD38 (**b**) and CD48 (**c**) mRNA in KDM6A KO and control H929 cells treated with GSK343 (4 μM) for 4 days. Data were normalized against GAPDH (mean ± SEM, n=3 biologically independent experiments). ***p*<0.01; ****p*<0.001 (two-sided student's *t* test). **d**, Western blotting of CD38 protein level in KDM6A WT and KO MM1S cells treated with Taze (5 μM) or GSK343 (4 μM) for 4 days. Three independent experiments were performed and similar results were obtained. **e**, Representative flow cytometry analysis of CD38 and CD48 expression in KDM6A KO and control H929 cells treated with GSK343 (4 μM) for 4 days. **f**,**g**, H3K27me3 ChIP-qPCR analysis at the CD38 (**f**) and CD48 (**g**) genes in KDM6A WT and KO MM1S cells (mean

± SEM, n=3 biologically independent experiments). **p<0.01; ***p<0.001 (two-sided student's *t* test). **h**, Representative flow cytometry analysis of CD38 expression in primary NK cells with Taze (5 μM) for 3 days. Source data are provided as a Source Data file.

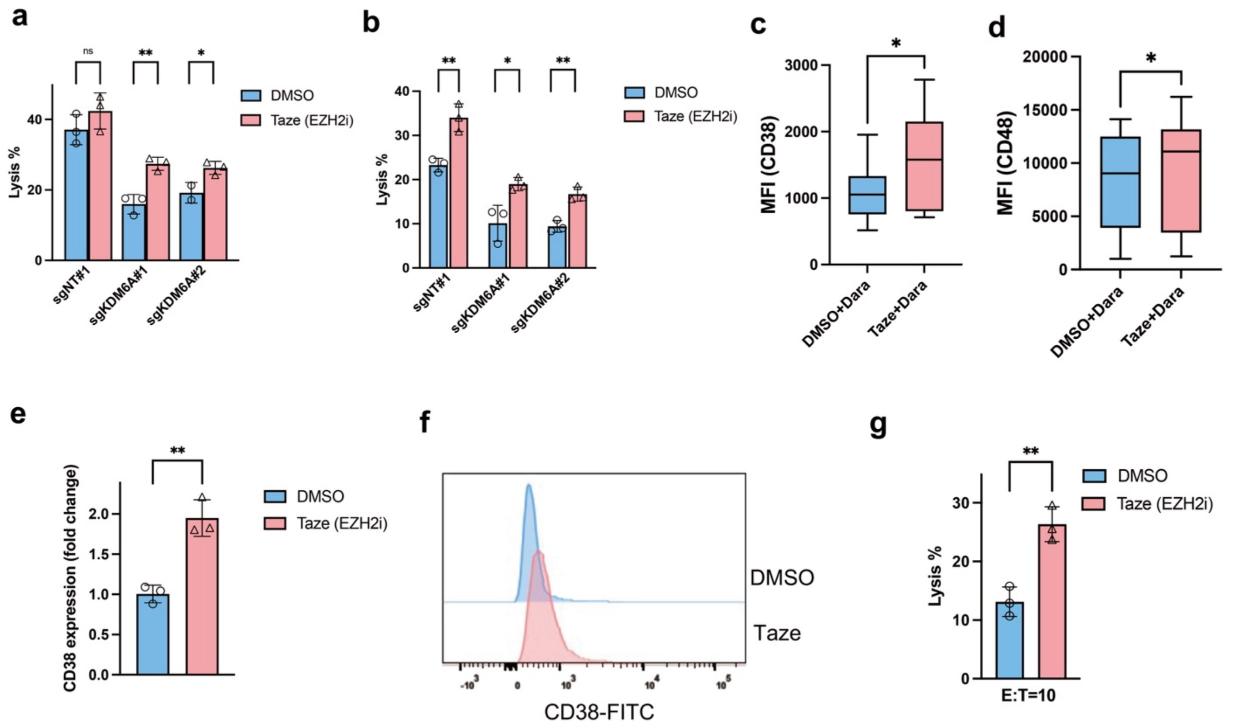

Supplementary Fig. 7. EZH2 inhibitors enhance Dara-mediated ADCC. a, *KDM6A* WT and KO H929 cells were treated with Taze (5 μM) for 4 days, then co-cultured with PBMC and Dara and subjected to ADCC assay (mean ± SEM, n=3 biologically independent experiments). ns, not significant; **p*<0.05 ***p*<0.01 (two-sided student's *t* test). **b**, *KDM6A* WT and KO MM1S cells were treated with Taze (5 μM) for 4 days, then co-cultured with PBMC and Dara and subjected to ADCC assay (mean ± SEM, n=3 biologically independent experiments). **p*<0.05 ***p*<0.01 (two-sided student's *t* test). **c**,**d**, The patients' MM cells were treated with DMSO or Taze (5 μM) for 4 days, followed by the addition of Dara and incubation for 12 hours. MFI of CD38 (**c**) and CD48 (**d**) were assessed by flow cytometry. (n=10 for each group). Box plots represent the median, 25th, and 75th percentiles, and whiskers represent the values of min and max. **p*<0.05 (two-sided paired student's *t* test). **e**, q-RT-PCR for CD38 mRNA in U266 cells treated with Taze (5 μM) for 4 days. Data were normalized against GAPDH (mean ± SEM, n=3

biologically independent experiments). $^{**}p<0.01$ (two-sided student's *t* test). **f**, Representative flow cytometry analysis of CD38 expression on U266 cells treated with Taze (5 μM) for 4 days. **g**, U266 cells were treated with Taze (5 μM) for 4 days, then co-cultured with primary human NK cells and Dara and subjected to ADCC assay (mean ± SEM, n=3 biologically independent experiments). $^{**}p<0.01$ (two-sided student's *t* test). Source data are provided as a Source Data file.
