# Supplementary material for: Epigenetic regulation of CD38/CD48 by KDM6A mediates NK cell response in multiple myeloma
Source: Nat Commun. 2024 Feb 14;15:1367. doi: 10.1038/s41467-024-45561-z (PMC10866908; doi:10.1038/s41467-024-45561-z)
Supplement: Supplementary file 3 — Description of Additional Supplementary Files [file 41467_2024_45561_MOESM3_ESM.pdf]

Description of Additional Supplementary Files

File Name: Supplementary Data 1
Description: 2CT genome-wide CRISPR screen

File Name: Supplementary Data 2
Description: Number of Genes Correlated with CYT

File Name: Supplementary Data 3
Description: CD38 low expression cells sorting CRISPR screen

File Name: Supplementary Data 4
Description: H929 KDM6A KO cells ATAC-seq_annotated.diff.peaks

File Name: Supplementary Data 5
Description: H929 KDM6A KO cells RNA-seq data

File Name: Supplementary Data 6
Description: Oligonucleotides
