# Supplementary material for: Epigenetic regulation of CD38/CD48 by KDM6A mediates NK cell response in multiple myeloma
Source: Nat Commun. 2024 Feb 14;15:1367. doi: 10.1038/s41467-024-45561-z (PMC10866908; doi:10.1038/s41467-024-45561-z)
Supplement: Supplementary file 10 — Reporting Summary [file 41467_2024_45561_MOESM10_ESM.pdf]

nature portfolio

Corresponding author(s): Kenneth C. Anderson

Last updated by author(s): Jan 22, 2024

Reporting Summary

Nature Portfolio wishes to improve the reproducibility of the work that we publish. This form provides structure for consistency and transparency in reporting. For further information on Nature Portfolio policies, see our Editorial Policies and the Editorial Policy Checklist.

Statistics

For all statistical analyses, confirm that the following items are present in the figure legend, table legend, main text, or Methods section.

n/a	Confirmed	
☐	☒	The exact sample size (*n*) for each experimental group/condition, given as a discrete number and unit of measurement
☐	☒	A statement on whether measurements were taken from distinct samples or whether the same sample was measured repeatedly
☐	☒	The statistical test(s) used AND whether they are one- or two-sided<br>*Only common tests should be described solely by name; describe more complex techniques in the Methods section.*
☐	☒	A description of all covariates tested
☒	☐	A description of any assumptions or corrections, such as tests of normality and adjustment for multiple comparisons
☐	☒	A full description of the statistical parameters including central tendency (e.g. means) or other basic estimates (e.g. regression coefficient) AND variation (e.g. standard deviation) or associated estimates of uncertainty (e.g. confidence intervals)
☐	☒	For null hypothesis testing, the test statistic (e.g. *F*, *t*, *r*) with confidence intervals, effect sizes, degrees of freedom and *P* value noted<br>*Give P values as exact values whenever suitable.*
☒	☐	For Bayesian analysis, information on the choice of priors and Markov chain Monte Carlo settings
☒	☐	For hierarchical and complex designs, identification of the appropriate level for tests and full reporting of outcomes
☒	☐	Estimates of effect sizes (e.g. Cohen's *d*, Pearson's *r*), indicating how they were calculated

Our web collection on statistics for biologists contains articles on many of the points above.

Software and code

Policy information about availability of computer code

Data collection	1. FACSDiva software (BD Biosciences)<br>2. IMAGE QUANT 800 (Cytiva)<br>3. QuantStudio Real-Time PCR Systems (Thermo Fisher)<br>4. SpectraMax M5 (Molecular Devices)<br>5. Living Image 4.2 software (PerkinElmer)
Data analysis	Statistical analysis were performed using PRISM 10.0 (Graphpad). Flow cytometry data was analyzed using FlowJo v10.<br>For ChIP-seq analysis: Trimmomatic v0.38, bowtie2, MACS2, IGV.<br>For RNA-seq data analysis: VIPER pipeline, STAR, limma.<br>For ATAC-seq analysis: Trimmomatic v0.38, bowtie2, Picard v2.18.26, MACS2 v2.1.2, R package Diffbind.

For manuscripts utilizing custom algorithms or software that are central to the research but not yet described in published literature, software must be made available to editors and reviewers. We strongly encourage code deposition in a community repository (e.g. GitHub). See the Nature Portfolio guidelines for submitting code & software for further information.

Data

Policy information about availability of data

All manuscripts must include a data availability statement. This statement should provide the following information, where applicable:

- Accession codes, unique identifiers, or web links for publicly available datasets
- A description of any restrictions on data availability
- For clinical datasets or third party data, please ensure that the statement adheres to our policy

CRISPR screen sequencing data, RNA-seq, ChIP-seq and ATAC-seq data generated in this study are deposited in the National Institute of Health Gene Expression Omnibus (GEO) database (GSE228771, https://www.ncbi.nlm.nih.gov/geo/query-/acc.cgi?acc=GSE228771).

Research involving human participants, their data, or biological material

Policy information about studies with human participants or human data. See also policy information about sex, gender (identity/presentation), and sexual orientation and race, ethnicity and racism.

Reporting on sex and gender	We do not do a specific analysis that considers sex as there is no expected sex-specific difference between the tumor sample of each group.
Reporting on race, ethnicity, or other socially relevant groupings	N/A
Population characteristics	Multiple Myeloma blood samples were collected from the patients with different stage of MM and different ages at Dana-Farber Cancer Institute in Boston, MA.
Recruitment	The human biospecimens were collected referred to BRISQ reporting guidelines.
Ethics oversight	This study was approved by the Institutional Review Board of the Dana-Farber Cancer Institute

Note that full information on the approval of the study protocol must also be provided in the manuscript.

Field-specific reporting

Please select the one below that is the best fit for your research. If you are not sure, read the appropriate sections before making your selection.

☒ Life sciences ☐ Behavioural & social sciences ☐ Ecological, evolutionary & environmental sciences

For a reference copy of the document with all sections, see nature.com/documents/nr-reporting-summary-flat.pdf

Life sciences study design

All studies must disclose on these points even when the disclosure is negative.

Sample size	No statistical method was used to predetermine sample size. The sample size of animal experiment was determined on the basis of our prior studies with similar experiment. For in vitro experiments, the sample size was determined on the basis of our prior studies and previous paper. The exact sample size has been displayed in the figures or stated in the figure legend or Method part.
Data exclusions	No data were excluded from analysis.
Replication	The data (except animal data) were obtained from at least 3 times repeated experiments with similar results. For the in vivo study, at least 5 animals were allocated in each group.
Randomization	The samples used in this study were randomly allocated into each group.
Blinding	For the in vivo study, the allocation of animal to each group, treatment and analysis were performed blindly by different researchers. For the in vitro study, cells were treated identically without prior designation.

Reporting for specific materials, systems and methods

We require information from authors about some types of materials, experimental systems and methods used in many studies. Here, indicate whether each material, system or method listed is relevant to your study. If you are not sure if a list item applies to your research, read the appropriate section before selecting a response.

Materials & experimental systems

n/a	Involved in the study
☐	☒ Antibodies
☐	☒ Eukaryotic cell lines
☒	☐ Palaeontology and archaeology
☐	☒ Animals and other organisms
☒	☐ Clinical data
☒	☐ Dual use research of concern
☒	☐ Plants

Methods

n/a	Involved in the study
☐	☒ ChIP-seq
☐	☒ Flow cytometry
☒	☐ MRI-based neuroimaging

Antibodies

Antibodies used	Antibodies were obtained as follows: KDM6A (no. 33510, Cell Signaling Technology, 1:1000), CD38 (no. 51000, Cell Signaling Technology, 1:1000), CD48 (no. 29499, Cell Signaling Technology, 1:1000), GAPDH (no. 5174, Cell Signaling Technology, 1:1000), H3K27me3 (no. 9733, Cell Signaling Technology, 1:1000), anti-rabbit immunoglobulin G (IgG), horseradish peroxidase (HRP)-linked Ab (no. 7074, Cell Signaling Technology, 1:2000), FITC anti-human CD38 (no. 356610, Biolegend, 1:20), APC anti-human CD38 (no. 356606, Biolegend, 1:20), FITC mouse IgG1 (no. 400110, Biolegend, 1:20), APC mouse IgG1 (no. 981806, Biolegend, 1:20), APC anti-human CD138 (no. 356506, Biolegend, 1:20), FITC anti-human IFN-[illegible] (no. 502506, Biolegend, 1:20), APC anti-human CD48 (no. 336714, Biolegend, 1:20), Alexa Fluor 488 anti-human MICA/B (no. 320912, Biolegend, 1:20), APC anti-human CD56 (no. 985906, Biolegend, 1:20), APC anti-human HLA-A,B,C (no. 311409, Biolegend, 1:20), APC anti-human CD253 (no. 308209, Biolegend, 1:20), FITC anti-human CD319 (no. 331817, Biolegend, 1:20), FITC anti-human CD155 (no. 337627, Biolegend, 1:20), PE anti-human ULBP-2/5/6 (no. FAB1298P, R&D systems, 1:20), BV421 anti-human CD178 (no. 306411, Biolegend, 1:20).
Validation	All the antibodies are from commercial sources and has been validated by the vendors and in numerous publications. The validation data are available on the manufacturer's website.

Eukaryotic cell lines

Policy information about cell lines and Sex and Gender in Research

Cell line source(s)	H929, MM1.S, U266, and human embryonic kidney (HEK) 293T cells were purchased from the American Type Culture Collection (ATCC). KMS-11 cells were purchased from Deutsche Sammlung von Mikroorganismen und Zellkulturen (DSMZ).
Authentication	All cell lines were verified by short tandem repeat (STR) DNA fingerprinting analysis (Molecular Diagnostic Laboratory, DFCI)
Mycoplasma contamination	All the cell lines were tested negative for mycoplasma using MycoAlert Mycoplasma Detection Kit (Lonza).
Commonly misidentified lines (See ICLAC register)	No commonly misidentified cell lines were used in this study.

Animals and other research organisms

Policy information about studies involving animals; ARRIVE guidelines recommended for reporting animal research, and Sex and Gender in Research

Laboratory animals	5-week-old CB17SC C.B-Igh-1 < b > /IcrTac-Prkdc < scid > female mice from Taconic Biosciences were used for Xenograft assay. All mice were housed in a pathogen-free environment in a temperature- and humidity-controlled environment on a 12 hrs light/dark cycle with sterilized and automated watering system at DFCI animal facility.
Wild animals	No wild animals were used in the study.
Reporting on sex	female. Sex was not considered in the study design.
Field-collected samples	No filed-collected samples were used for this study.
Ethics oversight	The experimental procedures and protocol (No: 03-043) were approved by the Institutional Animal Care and Use Committee (IACUC; DFCI).

Note that full information on the approval of the study protocol must also be provided in the manuscript.

Plants

Seed stocks	N/A
Novel plant genotypes	N/A

Authentication | N/A

ChIP-seq

Data deposition

☒ Confirm that both raw and final processed data have been deposited in a public database such as GEO.

☒ Confirm that you have deposited or provided access to graph files (e.g. BED files) for the called peaks.

Data access links
May remain private before publication.

https://www.ncbi.nlm.nih.gov/geo/query-/acc.cgi?acc=GSE228771

Files in database submission

GSM7136121 H929 cells, control, H3K27me3, CST
GSM7136122 H929 cells, KDM6A KO#1, Input
GSM7136123 H929 cells, KDM6A KO#1, H3K27me3, CST
GSM7136124 H929 cells, KDM6A KO#2, Input
GSM7136125 H929 cells, KDM6A KO#2, H3K27me3, CST

Genome browser session
(e.g. UCSC)

N/A

Methodology

Replicates

N/A

Sequencing depth

Sample FastQC TotalReads(M) MappedReads(M) UniqMappedReads(M) UniqLoc4M UniqLoc1read4M PBC
HK_1_H3K27me3 36 190.3 189.4 176.2 3.7 3.7 98.19
HK_1_Input 36 134.8 134.5 127.9 3.9 3.8 98.91
HK_2_H3K27me3 36 267.5 266.5 245.9 3.6 3.5 97.84
HK_2_Input 36 286.6 285.7 271.5 3.9 3.8 98.95
HNT_H3K27me3 36 120.1 119.6 111.2 3.8 3.7 98.16
HNT_Input 36 109.8 109.4 104.2 3.9 3.8 98.88

Antibodies

H3K27me3 (no. 9733, Cell Signaling Technology)

Peak calling parameters

The Peaks Summary table not only shows the statisics represented in the "Peak FoldChange" plots, but also give other statistics such as the FRiP score, number of DHS peaks, and peak characterization.

FRiP (or FRaction of reads In Peaks) score is calculated by first downsampling to 4 million reads, and aligning those reads to the genome. MACS is then used to call peaks on the resulting alignment (4M downsampled peaks). The FRiP is simply the fraction of th 4 million reads that fall within a peak-region.

DNAse hyper-sensitive (DHS) sites have been shown to be very active regions of the genome (ref). Using a list of known DHS regions (specific to each species), the DHS peaks represent the number of 4M downsampled peaks that intersect with DHS peaks.

The fraction of peaks that fall in promoter, exon, intron, and intergenic regions are also shown.

Run TotalPeaks FC>10 FC>20 FRiP DHS_peaks DHS_% Promoter Exon Intron Intergenic
HK_1_H3K27me3.rep1 106524 1718 134 32.9 4458 89.16 3.1 1.3 35.1 60.6
HK_2_H3K27me3.rep1 121076 1699 236 33.6 4400 88 2.8 1.1 34.4 61.7
HNT_H3K27me3.rep1 85785 2011 197 29.5 4411 88.22 3.5 1.5 35.9 59.1

Data quality

Run TotalPeaks FC>10 FC>20 FRiP DHS_peaks DHS_% Promoter Exon Intron Intergenic
HK_1_H3K27me3.rep1 106524 1718 134 32.9 4458 89.16 3.1 1.3 35.1 60.6
HK_2_H3K27me3.rep1 121076 1699 236 33.6 4400 88 2.8 1.1 34.4 61.7
HNT_H3K27me3.rep1 85785 2011 197 29.5 4411 88.22 3.5 1.5 35.9 59.1

Software

Low-quality reads and sequencing adapters were removed by Trimmomatic v0.38, and the qualified reads were mapped to the human genome (hg38) by bowtie2. Peak calling was performed by MACS252 and visualized in IGV. The intensity of peaks around the transcription start site (TSS) was calculated and shown by deeptools.

Flow Cytometry

Plots

Confirm that:

☒ The axis labels state the marker and fluorochrome used (e.g. CD4-FITC).

☒ The axis scales are clearly visible. Include numbers along axes only for bottom left plot of group (a 'group' is an analysis of identical markers).

☒ All plots are contour plots with outliers or pseudocolor plots.

☒ A numerical value for number of cells or percentage (with statistics) is provided.

Methodology

Sample preparation	MM cell lines were collected and washed with PBS and stained with Fixable Viability Dye eFluor 780 at 4°C for 30min to exclude dead cells. After the incubation, cells were washed with PBS and stained with conjugated primary antibodies or isotype control (IgG). Cells were then washed with 2% FBS containing PBS, and resuspended in Flow Staining Buffer. Cells were acquired in a BD LSRFortessa Flow Cytometer, and data were analyzed by FlowJo software.
Instrument	We used BD FACSCanto
Software	Flow cytometry data was collected using FACSDiva software (BD Biosciences). Flow cytometry data was analyzed using FlowJo v10.
Cell population abundance	N/A
Gating strategy	Cells were gated by FSC-A and SSC-A, ant then gated for single cells by FSC-A and FSC-H. Then cells were identified based on Fixable Viability Dye eFluor 780 and indicated primary antibodies staining.

☐ Tick this box to confirm that a figure exemplifying the gating strategy is provided in the Supplementary Information.
